# 

**Published:** 1858-07

**Authors:** 


### NOTES AND CORRESPONDENCE.

The Editor is anxious to give free scope to the expression of opinion, but will not hold himself responsible for the opinions of those of his contributors who attach their names to their papers.

We record with great pain the death of our esteemed friend and contributor, Dr. John Snow. Dr. Snow died, at 3 p.m. on Wednesday, June 16th, 1858, at his residence, 18, Sackville Street, after six days illness. Dr. Snow was but in the prime of life at the time of his decease. He was born on the 13th of March, 1813. His immense labours in the cause of sanitary science are well known to the readers of the Sanitary Review. But his estimable character, his innate love for science, his knowledge, can only be appreciated by those who had the pleasure of his intimate acquaintance. The relatives of Dr. Snow, knowing the earnest friendship which existed between him and the Editor of this Journal, have placed in the hands of the Editor the sheets of Dr. Snow's last contribution to medical science; the which, with a memoir, will appear in a few days.

The papers from Dr. Pickells of Cork and Dr. Foote of Constantinople will appear in our next number.

The conclusion of Mr. Cox's excellent paper on "Epidemics and their Everyday Causes" is in type, but is unavoidably postponed till next number.

Mr. Osborn will receive a private note.

### BOOKS RECEIVED.

A Bill to amend the Public Health Act, and to make further provision for the Local Government of Towns and Populous Districts. London: 1858. Presented by the Board of Health

Brown-Séquard (E., M.D.) Volume of his complete Physiological Works, collected and presented by the Author. 1858.

Davey (James G., M.D.) On the Ganglionic Nervous System. London: Churchill. 1858.

Davis (F. G., M.D.) Special Exercises in the Treatment of Diseases of the Lungs. New York: Hall and Clayton. 1858.

Dickins (Alfred L., C. E.) Report to the General Board of Health on the Townships of Dalton and Almondbury. London: 1858. Presented by the Board of Health.

Dunn (Robert, F.R.C.S.Eng.) An Essay on Physiological Psychology. London: Churchill. 1858.

Excelsior: or Murray's Royal Asylum Literary Gazette. Perth. January 1858.

Gant (Frederick J.) Evil Results of Overfeeding Cattle. A new inquiry, with engravings of the heart, lungs, &c., of the Diseased Prize Cattle lately exhibited by the Smithfield Cattle Club. London: Churchill. 1858.

Glaisher (James, F.R.S.) On the Meteorology of England during the Quarter ending March 31st. London: 1858.

Glaisher (James, F.R.S.) On the Meteorological and Physical Effects of the Solar Eclipse of March, 1858. London: 1858.

Guy (W. A., M.B.) The Mortality of the British Army. London: 1858.

Howe (S. G., M.D.) On the Causes of Idiocy. Edinburgh: Machlachlan and Stewart. 1858.

Lawrance (Richard Moore, M.D.) On Localized Galvanism applied to the treatment of Paralysis and Muscular contractions. London: Renshaw. 1858.

[A very interesting work, showing sound practical knowledge on the part of its author.]

Lee (Edwin, M.D.) The effect of Climate on Tuberculous Diseases. London: Churchill. 1858.

Lister (Joseph, F.R.C.S.) Spontaneous Gangrene, and the Causes of Coagulation of Blood in Diseases of the Blood-Vessels. Edinburgh: 1858.

McWilliam (J. O., M.D., F.R.S.) Statistical Account of the Health of the Waterguard and Waterside Officers of H.M. Customs during ten years ending December 31st, 1856. London: 1857.

Milner (W. R., Esq.) Meteorological Table for the year 1857, taken at Wakefield Prison.

Ogle (William, M.D.) Remarks on Provident Dispensaries. London: 1857.

Payne (M., M.D.) The Rights of Authors. Appendix to the Institutes of Medicine, by the same Author.

The Philanthropist and Social Science Gazette. London: May 1858.

Pinkerton (A. W. P., M.D.) The Spread of Cholera by Personal Communication. Edinburgh: Murray and Gibb. 1858.

Principles of Medical Reform. Circular. London: 1858.

Radcliffe (Charles Bland, M.D.) Epilepsy and other Convulsive Affections. London: Churchill. 1858.

Ranger (William, C.E.) Report to the General Board of Health on the Town of Lowestoft. London: 1858. Presented by the Board of Health.

Report of the Odontological Society of London, March 1858. London: Savill and Edwards. 1858.

Report (Second Annual) of the Medical Officer of Health, made to the Vestry of St. James's, Westminster, April 1858. Westminster: 1858.

Report of the Council of the British Meteorological Society. May 1857. London.

Report (Second) of the Commissioners of Her Majesty's Customs on the Customs. London: 1858

Report (Annual) of the National Vaccine Board. London: 1858. Presented by the Board of Health.

Returns of the expense of Printing, Postage, &c., of the Weekly Return of Newspaper of the Board of Health. London: 1858. Presented by the Board of Health

Roberton (John)! Additional Suggestions with a View to the Improvement of Hospitals for the Sick and Wounded. Manchester.

Robertson (W. Tindal, M.D.) Sanitary Science, its past and present state. London: Walton and Maberly. 1858.

[This admirable address will receive the notice it deserves in an ensuing number; it came too late for the present.]

Sims (J. Marion, M.D.) Silver Sutures in Surgery. Anniversary Discourse before the New York Academy of Medicine. New York: S. and W. Wood. 1858.

Snow (E. W., M.D.) Third Annual Report on the Births, Marriages, and Deaths in the City of Providence, for the year ending December 31st 1857. Providence: Knowles and Co. 1858.

Snow (E. W., M.D.) History of the Asiatic Cholera in Providence.

Transactions of the Social Science Association. London: 1858

Wells (T. Spencer, F.R.C.S.) On the Radical Cure of Reducible Inguinal Hernia. Dublin: H. M. Gill. 1858.

Westall (E.) Quarterly Mortality of Croydon, January 1st to March 31st, from 1848 to 1858 inclusive

---

### IN EXCHANGE.

Literary Gazette.
Psychological Journal.
British Medical Journal.
Ranking and Radcliffe's Abstract of the Medical Sciences.
Chemist.
Veterinarian.
Asylum Journal of Mental Science.
Quarterly Journal of the Chemical Society.
Scottish Review.
Edinburgh Medical Journal.
Glasgow Medical Journal.
Dublin Hospital Gazette.
American Journal of the Medical Sciences.
New York Journal of Medicine.
Montreal Medical Chronicle.
Charleston Medical Journal.
Gazette Médicale de Paris.
Aerzliches Intelligenz-Blatt.
Medical and Surgical Reporter.
Journal of Practical Medicine and Surgery.
Journal de Physiologie.
Gazette Médicale de l'Orient

*Is now Published, price* 16*s.*

### THE ASTLEY COOPER PRIZE ESSAY,

ON THE

### CAUSE OF THE

### COAGULATION OF THE BLOOD:

WITH A PRACTICAL APPENDIX.

BY

BENJAMIN W. RICHARDSON, M.D., L.R.C.P.,

PHYSICIAN TO THE ROYAL INFIRMARY FOR DISEASES OF THE CHEST.

---

### Contents:

CHAPTER I. Historical Memoranda on the supposed Causes of Coagulation of the Blood.

CHAPTER II.—Cause of the Phenomenon of Coagulation: present position of the Question.

CHAPTER III.—The Author's Personal Observations and Deductions on the Condition of the Blood in the Body under certain Physiological and Pathological States.

CHAPTER IV.—Experimental Inquiry into the Physical Agencies influencing the Coagulation of the Blood.

CHAPTER V.—Experimental Inquiry into the Chemical Agencies influencing the Coagulation of the Blood.

CHAPTER VI.—Experimental Inquiry into the Chemical Agencies influencing the Coagulation of the Blood (continued).

CHAPTER VII.—Phenomenon of the Buffy Coat.

CHAPTER VIII.—Summary of Conclusions and Propositions.

APPENDIX.

1.—Superalkaline Conditions of the Blood in Relation to Disease.
2.—Ammonia as an Exhalation from the Body.
3.—Experiments, shewing the Effect of Lactic Acid on Animal Bodies (with Coloured Plates, shewing the stages of Endocarditis).
4.—Deposition of Fibrin in the Heart and Blood-vessels during Life.
5.—The Alkalies and Acids as Remedial Agents.
6.—Application of Ammonia to the Experiment of Transfusion of Blood.
7.—On some Conditions of the Blood after Death, in relation to Medico-Legal Inquiries.
8.—List of Authors.

---

*The Essay is Illustrated with numerous Wood Engravings, and with Coloured Plates by Tuffen West.*

---

OPINIONS OF THE PRESS.

"We shall merely give an outline of what we may justly call Dr. Richardson's great discovery, feeling certain that all who desire to keep themselves up to the professional science of the day will study the work for themselves.....It is with extreme pride and satisfaction that we congratulate our countryman upon the appearance of his great work."—*Medical Times and Gazette.*

"We recommend to our readers a careful perusal of the whole treatise, which we look upon as not less remarkable for its high value as a model of logical research and a study for experimental physiologists, than it is memorable in the annals of our scientific literature for its luminous truths and satisfactory results."—*The Lancet.*

"The essay of Dr. Richardson affords an instructive lesson, as well as encouragement, to all who are engaged in investigating the phenomena of nature."—*British Medical Journal.*

---

London: JOHN CHURCHILL, New Burlington Street.

